# Excess Early Postnatal Weight Gain Leads to Increased Abdominal Fat in Young Children

**DOI:** 10.1155/2012/141656

**Published:** 2012-05-09

**Authors:** Annemieke M. V. Evelein, Frank L. J. Visseren, Cornelis K. van der Ent, Diederick E. Grobbee, Cuno S. P. M. Uiterwaal

**Affiliations:** ^1^Julius Center for Health Sciences and Primary Care, University Medical Center Utrecht, P.O. Box 85060, Utrecht, The Netherlands; ^2^Department of Vascular Medicine, University Medical Center Utrecht, P.O. Box 85500, Utrecht, The Netherlands; ^3^Department of Pediatric Pulmonology, Wilhelmina Children's Hospital, University Medical Center Utrecht, P.O. Box 85090, Utrecht, The Netherlands

## Abstract

*Background*. Increased childhood weight gain has been associated with later adiposity. Whether excess early postnatal weight gain plays a role in childhood abdominal fat is unknown. *Design*. In the ongoing Wheezing Illnesses Study Leidsche Rijn (WHISTLER), birth cohort weight and length from birth to age 3 months were obtained. In the first 316 five-year-olds, intra-abdominal and subcutaneous fat were measured ultrasonographically. Individual weight and length gain rates were assessed in each child. Internal *Z*-scores of weight for length gain (WLG) were calculated. Multiple imputation was used to deal with missing covariates. *Results*. Per-1-unit increase in *Z*-score WLG from birth to 3 months, BMI, waist circumference, and subcutaneous fat were significantly higher; 0.51 kg/m^2^, 0.84 cm, and 0.50 mm, respectively. After multiple imputation, a trend towards significance was observed for intra-abdominal fat as well (0.51 mm/SD). In the associations with 5-year adiposity, no interaction between postnatal *Z*-score WLG and birth size was found. *Conclusion*. Excess early postnatal weight gain is associated with increased general and central adiposity, characterized by more subcutaneous and likely more intra-abdominal fat at 5 years of age.

## 1. Introduction

Obesity is one of the major cardiovascular disease (CVD) risk factors and is a common health problem both in adults and in children [1]. Not only do obese children have a higher risk of becoming obese adults [2], childhood BMI is independently associated with later coronary heart disease as well [3]. Visceral fat in particular is related to an adverse metabolic profile [4] and increased CVD risk [5] through the production and secretion of metabolic active compounds like adipokines and cytokines, with effects on insulin sensitivity, lipid metabolism, and inflammation among others [6]. From a CVD prevention point of view, early prevention of intra-abdominal fat accumulation might help reducing the prevalence of type 2 diabetes mellitus and CVD. Identification of early life determinants of abdominal fat distribution is therefore important.

 Growth patterns in early life have been suggested to be important for later fat distribution. Several studies determined an association between weight gain in the first 2-3 years of life and central adiposity, mostly assessed by larger waist circumference, in both adulthood [7] and early childhood [8–13]. Imaging techniques such as magnetic resonance imaging [7], ultrasonography [10, 11], or computed tomography have seldom or never been used to assess the association between postnatal growth and later central adiposity. While in these studies weight gain in the first years of life was studied, the importance of particularly the first 3 months of life in the development of central adiposity was addressed in other studies in children [14] and adults [15–17]. Weight gain in the first 3 postnatal months is thought to comprise predominantly fat mass accumulation; fat mass as percentage of body weight increases until 3–6 months of age, followed by a gradual decline [18]. Most of the previous studies did not account for length gain in infancy, while weight gain relative to length gain might reflect adiposity better than weight gain alone [19, 20]. Whether particularly rapid weight gain for length gain in the first 3 postnatal months, so excess weight gain relative to length gain, is related to differences in intra-abdominal fat, measured ultrasonographically, in childhood remains unknown.

 We set out to measure intra-abdominal and subcutaneous fat ultrasonographically in healthy 5-year-old children, to study whether differences in growth in the first 3 postnatal months, in particular increased weight gain accounting for length gain, are associated with increased central adiposity in early childhood. 

## 2. Methods

### 2.1. Design and Study Population

**Figure 1:**
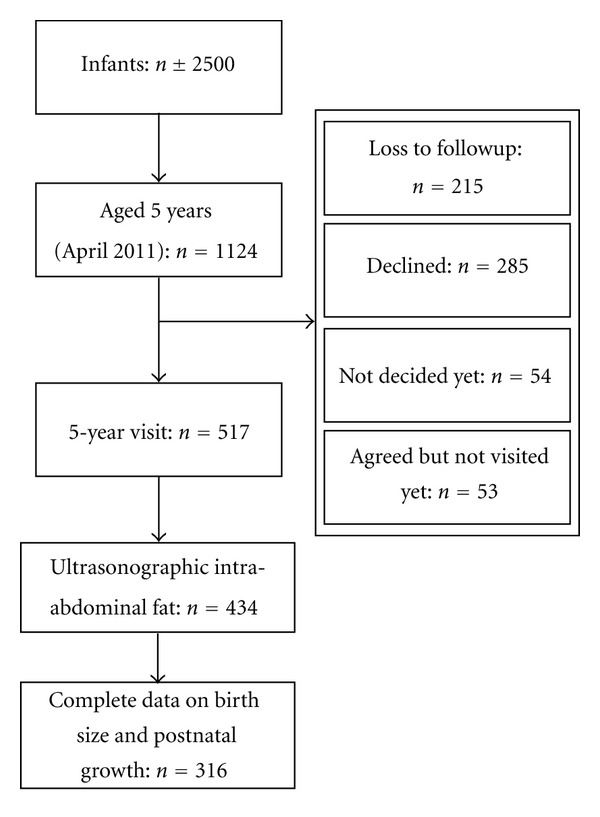
Overview of the study population.

The present study is part of the Wheezing Illnesses Study LEidsche Rijn (WHISTLER) study, an ongoing population-based birth cohort on determinants of wheezing illnesses, initiated in 2001 [21]. Healthy newborns in Leidsche Rijn, a new residential area near Utrecht city, were enrolled. Exclusion criteria were gestational age < 36 weeks, major congenital abnormalities, and neonatal respiratory disease. Currently, over 2500 infants have been included. In 2007 the study was extended for cardiovascular research questions (WHISTLER-Cardio). All five-year-olds (*n* = 1124 on April 26th, 2011) were invited according to the last-known telephone number and address, for follow-up measurements. 215/1124 (19%) subjects were lost to follow-up, due to incorrect telephone numbers and addresses, and 54/1124 (5%) were not yet contacted. Of the remaining 855 subjects, 285 (33%) declined to take part and 570 (67%) were willing to participate of whom 517 were measured before April 26th 2011. Abdominal ultrasonography was performed and intra-abdominal fat and subcutaneous fat were measured successfully in 434/517 (84%) and 463/517 (90%) subjects, respectively. In the remaining participants the focus was solely on respiratory measures. Complete data on postnatal weight gain relative to length gain and abdominal fat distribution were available for 360/517 children (70%), and in addition on birth size for 316/517 (61%). An overview of the study population is presented in Figure 1.

WHISTLER-Cardio was approved by the paediatric Medical Ethical Committee of the University Medical Center Utrecht. Written informed parental consent was obtained.

### 2.2. Neonatal Visit and Follow-Up in Infancy

Parents visited the clinic when their offspring was approximately four weeks of age for lung function measurements, not further described here [22]. Birth weight in 359/360 (99.7%) children and birth length in 317/360 (88%) children were measured standardized in the hospital or by midwifes using a standard electronic scale and an infant stadiometer. Parents were asked to report these measures in a questionnaire. This questionnaire inquired for maternal age at childbirth and gestational age among others as well. In the Netherlands infants regularly visit Child Health Care Centers for standardized weight and length measurements. We asked parents to report these measurements in monthly questionnaires for a period of 12 months. The type of infant feeding was reported monthly as well [23]. Parents were instructed to answer “exclusively breastfed” if the only milk their infant had received during the month of interest was breast milk. Data on parental characteristics, like parental BMI, ethnicity and level of education among others, were obtained from the linked database of the Utrecht-Health-Project (UHP), a large health monitoring study in Leidsche Rijn [24].

### 2.3. Follow-Up Measurements

Methods of the follow-up measurements have been described previously [25]. Children were reinvited to visit the outpatient clinic at 5 years of age. Weight, height, and waist circumference were measured with the participants wearing indoor clothes without shoes. Standing with the feet slightly spread waist circumference was measured in duplicate at the level midway the lowest rib border and the iliac crest and hip circumference at the widest level over the major trochanter to the nearest mm. Body mass index (BMI, kg/m^2^) was calculated. In addition, intra-abdominal and subcutaneous fat were measured using ultrasound according to a previously described procedure [26, 27] with a Picus Pro system (Esaote, Italy), using a CA 421 convex transducer. For intra-abdominal fat, the distances between the posterior edge of the abdominal muscles and the lumbar spine were measured using electronic callipers. Distances were measured from 3 different angles: medial, left, and right lateral, with the transducer placed longitudinally on a straight line drawn between the left and right midpoint of the lower rib and iliac crest. Measurements were performed at the end of a quiet expiration. The average distance was calculated from the three angles. Placing the probe transversely at the level of the umbilicus subcutaneous fat was measured with electronic callipers, from the external face of the rectus abdominis mucle (linea alba) to just below the skin. The measurement was repeated three times and the average was used for analysis. For all measurements, minimal pressure was applied to eliminate manual compression of tissue. All measurements were performed by two of the investigators and a trained research nurse all blinded to infancy weight and length gain. Intraclass correlation coefficients (ICC) based on measurements by one observer in 10 and, respectively, 11 subjects on 2 different occasions for intra-abdominal and subcutaneous fat were 0.67 and 0.96, respectively. ICCs for subcutaneous fat on the three measurements per child on the same occasion were 0.94, 0.94, and 0.97 for the three observers.

In addition, information on child and parental characteristics, like smoking habits among others, with respect to the previous years was collected by questionnaires.

### 2.4. Infancy Growth

We used weight gain rate, length gain rate, and particularly weight gain rate adjusted for length gain rate as measures of growth in the first 3 postnatal months. These measures were studied separately, as length gain might reflect lean mass accumulation, while weight gain, and particularly weight gain accounting for length gain, might reflect fat mass accumulation.

For each child with at least two anthropometric measurements available in the first 3 postnatal months, weight gain and length gain rates were estimated using the monthly anthropometrics, to reduce an effect of measurement error and individual variation over time (regression to the mean). Since the number of weight and length measurements (median count: 3) and the age at which these were measured differed per child, the association between age, and both weight and length was assessed using linear mixed modelling, allowing for individual variation in birth weight or height and growth rate. Subsequently, to obtain weight gain rates for each child individually, linear regression modelling on the predicted weight and length values of the linear mixed model was performed stratified by child. The same steps were taken to assess individual length gain rates. Because of the small time window, weight and length gain rate were assumed constant in the first 3 postnatal months. WLG was assessed by deriving *Z*-scores internal to our study population for weight gain, conditioned on length gain, by using the standardized residuals from the linear regression model with weight gain as the dependent variable and length gain as the independent variable. A *Z*-score of +1 SD WLG indicates that the weight gain of a certain child is one standard deviation larger than the mean weight gain in the population based on the length gain of that child. Furthermore, size at birth was assessed by calculating internal *Z*-scores for birth weight, adjusted for birth length, gestational, age and gender.

### 2.5. Data Analysis

Means and dispersion measures of parent and child characteristics were calculated by tertiles of *Z*-score WLG. Differences by tertiles of *Z*-score WLG were tested using analysis of variance, or Kruskal Wallis test in case of skewed data, for continuous variables and Chi-squared tests or Fisher's exact tests for frequencies (Table 1).

For analyzing the associations between postnatal growth and fat distribution we used generalized linear modelling. BMI, waist circumference, intra-abdominal fat, and subcutaneous fat were used as dependent variables in separate models. Weight gain rate, length gain rate, and *Z*-score WLG were used as independent variables in separate models. After univariable analysis, adjustments for age at follow-up and gender were made. In the analyses of intra-abdominal and subcutaneous fat we additionally adjusted for observer to reduce the possibility for observer bias. Moreover, adjustments for current height were made, as achieved height can be considered as confounder when studying the association between early weight gain and WLG and fat distribution. Parental characteristics, like smoking and BMI, and infant nutrition, were not considered as confounders, as to our view these factors might explain the associations between growth and later fat distribution and might therefore be in the causal pathway.

Since an association between postnatal growth and fat distribution might be modified by fetal growth as well, we analyzed whether interaction on an additive scale was present between birth size, as proxy for fetal growth, and early postnatal growth on fat distribution.

Data on birth size was missing in 44 children due to missing birth length. We therefore imputed birth length using multiple imputation technique in SPSS version 17.0 for Windows and repeated the analyses on the 10 imputed datasets as sensitivity analysis.

All results are expressed as linear regression coefficients with 95% confidence intervals (95%-CI) and corresponding *P* values. Statistical significance was considered reached at *P*_2-sided_ < 0.05.  All analyses were performed with SPSS version 17.0 for Windows.

## 3. Results

Median BMI, waist circumference, subcutaneous fat, and mean intra-abdominal fat were 15.1 kg/m^2^ (interquartile range (IQR): 14.3–16.1), 52.5 mm (IQR: 50.5–54.8), 6.3 mm (IQR: 5.0–7.9), and 36.4 ± 6.5 mm, respectively. Children with higher postnatal WLG were relatively thin at birth. No differences in infant feeding type and parental characteristics were determined across growth tertiles, except for parental smoking habits and maternal gestational diabetes: parents of children with lower WLG smoked more often in the 5 years after birth and all three infants of the mothers who have had gestational diabetes belonged to the highest *Z*-score WLG tertile. Postnatal WLG was positively associated with weight and height of the children at the follow-up visit at 5 years of age (Table 1).

 In Table 2 the associations between early postnatal growth and body fat distribution at the age of 5 years are shown. After correcting for confounders, size at birth was positively associated with BMI and waist circumference, but not with intra-abdominal and subcutaneous fat. With respect to postnatal growth, both weight gain and *Z*-score WLG were significantly positively associated with BMI, waist circumference, and subcutaneous fat at the age of 5 years. No significant associations between both weight gain and WLG with intra-abdominal fat were observed. Length gain was associated with none of the measures of body fat distribution. Size at birth did not modify the associations between postnatal growth and body fat distribution.

 After multiple imputation of birth length, to have complete data on size at birth for all 360 children in which an ultrasound was successfully performed and growth data was available, the associations between postnatal WLG and BMI, waist circumference, and subcutaneous fat strengthened. Regarding intra-abdominal fat, a trend towards significance was observed: per 1 unit increase in *Z*-score WLG, intra-abdominal fat was 0.51 mm higher (95%-CI: −0.14, 1.2, *P* value: 0.12). Again, no interaction between size at birth and postnatal growth on body fat distribution was present.

## 4. Discussion

The present study contributes to current knowledge in showing that excess weight gain relative to length gain in the first 3 months after birth is associated with central adiposity. Strong associations with BMI, waist circumference, and abdominal subcutaneous fat were observed. In addition, these results suggest an increase in intra-abdominal fat as well.

 Some remarks have to be made. WHISTLER is a population-based cohort and families with a healthier lifestyle might be more willing to participate. Moreover, children with follow-up measurements tended to have a higher birth weight and were larger at birth (data not shown) compared to those lost to follow-up. Since data on birth size was not available for all children, birth length was imputed using multiple imputation. Missing birth length was associated with lower gestational age and the imputed birth lengths were smaller than those nonimputed. Multiple imputation strengthened the observed associations between postnatal growth and adiposity measures in young childhood, indicating an effect of selection on the results from the complete case analyses. We consider our study population to be representative of a healthy pediatric population, as the mean weights and lengths in infancy are in line with the WHO growth charts [28]. We derived internal *Z*-scores for weight gain rate conditioned on length gain rate, as references values for comparable *Z*-scores are not available. Since data on fetal growth have not been collected as part of the present study, birth size was used as proxy. Birth weight is determined by gestational age, growth potential, and intrauterine environment. Therefore, birth weight adjusted for birth length and gestational age might be a better indicator of intrauterine exposures. Besides age, gender, current height, and sonographer, we did not consider other factors, like parental BMI, infant feeding, tobacco smoke exposure among others as confounders, as these factors might precede the studied association and might therefore be in the causal pathway. Whether or not to adjust for achieved height is topic of discussion [29, 30]. However, when comparing absolute fatness of individuals of different heights, adjustment for height is indicated [19, 20]. Since the ultrasonography was performed blinded to infancy characteristics, the probability of information bias to have occurred is negligible. Additionally, we adjusted for sonographer, to further rule out the possibility of observer bias. Although CT is currently the most accurate method to measure intra-abdominal fat [31], ultrasonography is a good, child friendly, and practical alternative in population studies, which has been shown to correlate well with results from CT and MRI [26]. When the data collection of the present study started, we chose to use ultrasonographically measured intra-abdominal depth to assess visceral fat [26, 27]. More recently, validation studies of this method in children provided conflicting results [32, 33] and another ultrasonographic method for measuring intra-abdominal fat has been described [34, 35] and validated in infants [36]. Although we previously found an association between intra-abdominal depth and vascular characteristics in the 5-year-olds of our study population as well [25], it should be kept in mind that consensus regarding the best, feasible method for measuring intra-abdominal fat in children, has not yet been reached.

Previously, associations between weight gain in the first 2 years of life and childhood BMI [8, 9], waist circumference [8, 12, 13], intra-abdominal fat [11], and both subcutaneous and preperitoneal fat [10] have been described. In the present study, we focused on excess weight gain relative to length gain the first 3 postnatal months, as the importance of particularly this period in the development of increased waist circumference in adulthood [15–17] and childhood [14] was addressed in previous studies. Associations between excess early postnatal weight gain and BMI, waist circumference, and abdominal subcutaneous fat were indeed observed in this study, and some evidence for an association with intra-abdominal fat was found as well. The difference in precision of the ultrasonographic fat measurements might explain why the association with intra-abdominal fat was less pronounced than the association with subcutaneous fat.

The observed differences in adiposity associated with excess weight gain in early infancy might be explained by increased accumulation of fat mass in the early postnatal period. Weight gain for length gain in the first months after birth primarily reflects accumulation of fat mass, since fat mass increases from approximately 13% of total body weight to 31% between 0.5 and 3–6 months of age [18]. According to the “developmental origins of human health and disease” hypothesis [37, 38] increased postnatal weight gain for length gain might be a reflection of metabolic programming following a period of relative growth impairment. However, in the present study no interaction between postnatal weight gain for length gain and size at birth was observed in the associations with adiposity at 5 years of age. This indicates that the association between increased weight gain for length gain and fat distribution is not only present for those infants who were growth restricted in pregnancy, but also for infants with normal fetal growth.

We found some evidence for an association between excess early postnatal weight gain and intra-abdominal fat. Besides these differences in quantity of intra-abdominal fat, the previously observed adverse metabolic consequences later in life associated with increased postnatal growth [15], might be further explained by differences in adipose tissue function. Adipose tissue dysfunction might be even more important than quantity alone in the development of CVD [6]. Further research is needed to study whether excess weight gain in early infancy leads to changes in adipose tissue functioning in early childhood.

While the observed differences in fat distribution are relatively small from an individual perspective, they are relevant from a population perspective. However, we should be careful in extrapolating these finding to growth interventions, as rapid postnatal growth might have benefits as well [39–41]. Moreover, to date it remains unknown how to modify infant growth in a way that avoids adverse outcomes. Based on current knowledge, prevention of known causes of impaired fetal growth and rapid postnatal growth, such as maternal smoking during pregnancy [42], is indicated.

In conclusion, variations in postnatal growth are associated with abdominal fat in early childhood. Over the whole range of birth size, excess early postnatal weight gain relative to length gain is associated with increased general and central adiposity, characterized by higher BMI, larger waist circumference, more abdominal subcutaneous fat, and likely more intra-abdominal fat at 5 years of age.

## Figures and Tables

**Table 1:** Associations between child and parental characteristics and tertiles of *Z*-score weight gain for length gain rate in the first 3 postnatal months.

	Tertiles of weight gain rate for length gain rate in the first 3 months after birth			
	1st *Z*-score WLG tertile (mean : −1.1)	2nd *Z*-score WLG tertile (mean : −0.049)	3rd *Z*-score WLG tertile (mean : 1.1)	
	*N* = 105	*N* = 106	*N* = 105	*P* value
Child characteristics				
*Infancy*				
Gender (% boys)	53	45	48	0.49^a^
Gestational age (days)^#^	282 (275–288)	279 (271–286)	281 (274–286)	0.07^c^
Birth weight (grams)	3564 ± 46	3430 ± 46	3642 ± 46	0.005^b^
Birth length (cm)	50.7 ± 0.22	50.7 ± 0.22	51.9 ± 0.22	<0.001^b^
*Z*-score birth size (SD)	0.18 ± 0.097	−0.14 ± 0.096	−0.044 ± 0.097	0.056^b^
Mean weight gain (g/day)	24.6 ± 0.26	28.0 ± 0.26	32.1 ± 0.26	<0.001^b^
Mean length gain (mm/day)	1.1 ± 0.005	1.1 ± 0.005	1.1 ± 0.005	0.95^b^
Breastfeeding (% ever)	79	79	80	0.98^a^
Exclusive breastfeeding (days)^#∗^	66 (26–127)	72 (28–136)	79 (40–145)	0.26^c^
*Childhood*				
Age at 5 years visit (years)^#^	5.4 (5.2–5.5)	5.3 (5.2–5.4)	5.3 (5.2–5.4)	0.16^c^
Weight (kg)	19.4 ± 0.26	19.8 ± 0.26	21.6 ± 0.26	<0.001^b^
Height (cm)	114.2 ± 0.45	114.3 ± 0.45	116.7 ± 0.45	<0.001^b^
BMI (kg/m^2^)^#^	14.8 (14.0–15.4)	14.9 (14.0–16.1)	15.6 (14.8–16.6)	<0.001^c^
Waist circumference (mm)^#^	52.2 (50.2–53.9)	52.0 (49.9–54.1)	54.0 (51.5–56.3)	<0.001^c^
Systolic blood pressure (mmHg)	105 ± 0.75	105 ± 0.76	106 ± 0.76	0.46^b^
Diastolic blood pressure (mmHg)	55 ± 0.68	56 ± 0.68	55 ± 0.68	0.60^b^

Parental characteristics				
Maternal age at childbirth (years)	32.5 ± 0.34	32.4 ± 0.34	32.1 ± 0.34	0.70^b^
Maternal BMI (kg/m^2^)	25.4 ± 0.44	24.8 ± 0.44	24.9 ± 0.43	0.58^b^
Paternal BMI (kg/m^2^)	25.6 ± 0.34	25.6 ± 0.32	25.6 ± 0.34	0.99^b^
Maternal gestational diabetes (%)	0	0	3	0.11^d^
Maternal smoking-prenatal (%)	6	6	1	0.13^a^
Parental smoking-postnatal (%)				0.039^a^
Neither one of the parents	62	70	79	
One parent	31	20	19	
Both parents	7	10	2	
Socio economic status (% high educated)	79	76	69	0.26^a^
Maternal ethnicity (% western)	93	91	92	0.85^a^

Values are means with standard errors in case of continuous variables and percentages in case of frequencies. In case of skewed data (^#^), medians with interquartile range were presented.

^#^Not normally distributed.

*Within those ever breastfed.

^a^Chi-square test.

^b^ANOVA.

^c^Kruskall Wallis test.

^d^Fisher's exact test.

**Table 2:** Associations between both size at birth and early infancy growth parameters and fat distribution indices at 5 years.

	Body mass index (kg/m^2^)		Waist circumference (cm)		Intra-abdominal fat (mm)		Subcutaneous fat (mm)	
	Linear regression coefficient (95%-CI)	*P* value	Linear regression coefficient (95%-CI)	*P* value	Linear regression coefficient (95%-CI)	*P* value	Linear regression coefficient (95%-CI)	*P* value
	*N* = 316		*N* = 314		*N* = 316		*N* = 315	
*Z*-score birth size*								
Crude	0.24 (0.080–0.39)	0.003	0.42 (0.007–0.83)	0.046	0.081 (−0.64–0.81)	0.83	0.15 (−0.21–0.51)	0.42
Model 1	0.24 (0.084–0.40)	0.003	0.45 (0.040–0.85)	0.031	0.048 (−0.67–0.77)	0.90	0.16 (−0.20–0.51)	0.38
Model 2			0.40 (0.029–0.77)	0.034	0.050 (−0.67–0.77)	0.89	0.13 (−0.21–0.48)	0.45
Model 3					0.12 (−0.54–0.78)	0.72	0.14 (−0.20–0.49)	0.42

Weight gain (gr/day)								
Crude	0.13 (0.089–0.16)	<0.001	0.34 (0.24–0.43)	<0.001	0.16 (−0.018–0.34)	0.078	0.096 (0.009–0.18)	0.031
Model 1	0.13 (0.087–0.16)	<0.001	0.33 (0.23–0.43)	<0.001	0.11 (−0.075–0.30)	0.24	0.16 (0.071–0.25)	<0.001
Model 2			0.21 (0.11–0.31)	<0.010	0.14 (−0.062–0.34)	0.18	0.10 (0.004–0.20)	0.040
Model 3					0.12 (−0.067–0.30)	0.21	0.10 (0.004–0.19)	0.041

Length gain (mm/day)								
Crude	0.79 (−2.6–4.2)	0.65	8.8 (0.005–17.6)	0.050	3.0 (−12.3–18.3)	0.70	−1.3 (−8.9–6.2)	0.73
Model 1	0.48 (−2.9–3.8)	0.78	7.7 (−1.0–16.4)	0.083	2.1 (−13.2–17.3)	0.79	−0.50 (−8.0–7.0)	0.90
Model 2								
Model 3					1.5 (−12.5–15.5)	0.84	−0.55 (−8.0–6.9)	0.89

*Z*-score weight for length gain**								
Crude	0.51 (0.37–0.66)	<0.001	1.2 (0.82–1.6)	<0.001	0.43 (−0.29–1.1)	0.25	0.68 (0.33–1.0)	<0.001
Model 1	0.51 (0.37–0.66)	<0.001	1.2 (0.83–1.6)	<0.001	0.42 (−0.29–1.1)	0.25	0.68 (0.34–1.0)	<0.001
Model 2			0.84 (0.46–1.2)	<0.001	0.49 (−0.26–1.2)	0.20	0.50 (0.15–0.85)	0.006
Model 3					0.44 (−0.24–1.1)	0.21	0.50 (0.15–0.85)	0.005

A *Z*-score of +1 SD WLG indicates that the weight gain of a certain child is one standard deviation larger than the mean weight gain in the population based on the length gain of that child.

*Birth weight adjusted for birth length, gestational age and gender.

**Weight gain rate adjusted for length gain rate and gender.

All results are linear regression coefficients with 95%-confidence intervals.

Model 1: adjusted for age and gender.

Model 2: adjusted for age, gender and current height (the analyses with rate of length gain are not adjusted for current height).

Model 3: adjusted for age, gender, current height and observer (for intra-abdominal fat and subcutaneous fat).
